# Characterization of a Commercial Ionization Chamber Array With Scanned Proton Beams for Applications in MRI‐Guided Proton Therapy

**DOI:** 10.1002/mp.17875

**Published:** 2025-05-13

**Authors:** Benjamin Gebauer, Sebastian Gantz, Daniela Kunath, Aswin Hoffmann, Jörg Pawelke, Felix Horst

**Affiliations:** ^1^ OncoRay ‐ National Center for Radiation Research in Oncology Faculty of Medicine and University Hospital Carl Gustav Carus Technische Universität Dresden and Helmholtz‐Zentrum Dresden‐Rossendorf Dresden Germany; ^2^ Helmholtz‐Zentrum Dresden‐Rossendorf Institute of Radiooncology ‐ OncoRay Dresden Germany; ^3^ Department of Radiotherapy and Radiation Oncology Faculty of Medicine and University Hospital Carl Gustav Carus Technische Universität Dresden Dresden Germany

**Keywords:** ionization chamber array, MRI‐guidance, proton therapy

## Abstract

**Background:**

The integration of MRI‐guidance and proton therapy is a current research topic. Proton therapy with the patient being placed inside an in‐beam MR scanner would require the presence of its static magnetic ($B_{0}$) field to be taken into account in dose calculation and treatment planning. Therefore, dosimetric tools are needed to characterize dose distributions in presence of the $B_{0}$ field of the MR scanner. Furthermore, patient‐specific quality assurance (QA) and treatment plan verification measurements should also be performed within the magnetic field.

**Purpose:**

In this work, the PTW Octavius 1500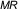 ionization chamber array was characterized experimentally and tested for its suitability as a dosimetric tool for beam characterization and QA in MRI‐guided proton therapy.

**Methods:**

The dose rate response, response homogeneity and effective measurement depth of the detector were determined in experiments with scanned proton beams delivered by a horizontal beamline at OncoRay, Dresden. A patient‐specific QA test including gamma analysis was performed for a realistic proton patient treatment plan at two different distances from the beam nozzle. In addition, experiments were performed in a 0.32T in‐beam MR scanner. These included measurements of square reference scanning patterns at different proton energies as well as measurements of a two‐field patient treatment plan at different water equivalent depths.

**Results:**

The dose rate response was found to be linear up to 80Gy/min. The effective measurement depth was determined to be 8.1±0.2mm. The response homogeneity was found to be suitable for the verification of proton treatment plans. The patient‐specific QA test without magnetic field was satisfactory and also the measurements inside the 0.32T in‐beam MR scanner provided reasonable results. Their comparison allowed an assessment of the magnetic field effects on the dose distributions.

**Conclusions:**

Concluding from these tests, the Octavius 1500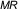 was found to be suitable for use as a dosimetric tool in MRI‐guided proton therapy.

## 1 INTRODUCTION

A recent development in radiotherapy that is being investigated worldwide is the integration of magnetic resonance imaging (MRI) guidance.^1^ While MRI‐linacs integrating photon therapy with onboard MRI‐guidance^2^ are nowadays available as commercial products from several vendors and are being installed at an increasing number of clinics worldwide, there is currently no ready solution for MRI integration with proton therapy available. In‐room image guidance for proton therapy is typically based on x‐ray and computed tomography (CT) systems, which often do not provide a sufficient soft‐tissue contrast for accurate target volume delineation and cause additional imaging dose to be administered to the patient. For this reason, and also because of the higher sensitivity to anatomical changes compared to photons, proton therapy may also benefit greatly from integration with MRI (magnetic resonance integrated proton therapy – MRiPT).^3^, ^4^ A major difficulty as compared to MRI‐guided photon therapy is that protons, being positively charged particles, are deflected in the static magnetic field of the MR scanner while at MRI linacs only the secondary electrons are affected.^5^ In proton therapy, however, the magnetic field induced beam deflection can in principle be easily compensated for by the pencil beam scanning (PBS) magnets.^6^, ^7^ This requires the implementation of the Lorentz force in the dose calculation engine of the treatment planning system, which is subject of ongoing research.^8^, ^9^, ^10^, ^11^

The University Proton Therapy Dresden (UPTD) at the University Hospital Carl Gustav Carus and OncoRay, Dresden, treats patients routinely in a treatment room equipped with a rotating gantry. An experimental room used for proton therapy research is located adjacent to the gantry room. First tests at the OncoRay experimental room with a 0.22 T prototype in‐beam MR scanner^12^, ^13^ have proven the technical feasibility of combining MRI and proton therapy. Due to the use of a low field MR scanner the beam deflection is reduced. After these successful tests the first prototype scanner was succeeded by a next generation prototype, a 0.32T MR scanner (MRJ3300, ASG Superconductors, Genova, Italy) housed in a walk‐in Faraday cabin mounted on an air‐cushion platform. This scanner would allow for a first‐in‐human application to prove the clinical feasibility and safety of in‐beam MRI‐guided proton therapy. In addition to ongoing developments and research in fields such as patient positioning, image processing and registration, a particularly important topic is proton dose calculation and treatment planning in the presence of the in‐beam MR scanner's magnetic field. The dose calculation algorithms that are currently under development require accurate input data for commissioning and their prediction of the proton trajectories through the magnetic field needs to be validated against dosimetric data. Such dose measurements must be performed inside the MR scanner and an active detector with 2D information would be highly preferable.

Another relevant topic when working towards the first treatment of patients inside the in‐beam MR scanner with protons is the development and implementation of dosimetric quality assurance (QA) procedures for this specific purpose. Besides machine QA^14^ and geometric alignment tests, one of the most important QA tasks in proton therapy is the individual treatment plan verification (*patient‐specific QA*).^15^ Patient‐specific QA verifies on one hand the performance of the beam application system and on the other hand the dose calculation accuracy of the treatment planning system. This is very important for MRiPT because the beam deflection in the transverse MR magnetic field adds a new level of complexity to the dose calculation and treatment plan optimization.

The absolute dose verification at selected points in a water phantom is possible also in the framework of MRiPT if chamber specific magnetic field correction factors are applied.^16^, ^17^ Besides absolute dosimetry checks, an important test for patient‐specific QA is the measurement of relative 2D dose distributions at different depths, for which at UPTD a scintillating screen detector (LynxPT, IBA Dosimetry, Schwarzenbruck, Germany) is the standard device. However, in tests performed in the 0.32T transverse $B_{0}$ field of our in‐beam MR scanner together with the manufacturer of the LynxPT, the camera system inside the device turned out to be malfunctioning and as a result no measurements were possible. Therefore, an alternative detector had to be identified that could be used for the measurement of 2D dose distributions inside our in‐beam MR scanner.

2D ionization chamber arrays have been shown to be suitable for the dosimetric verification of treatment plans for scanned proton beam delivery^18^, ^19^, ^20^ despite their relatively low spatial resolution (i.e., common models have chamber spacing in the order of centimeters). Their sensitivity for detecting 2D dose irregularities is comparable to the IBA Lynx PT detector.^21^ Therefore, the aim of this study was to test the Octavius 1500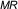 detector (PTW Freiburg, Freiburg, Germany), an ionization chamber array originally designed for dosimetric QA tasks performed in magnetic fields of MRI‐linacs, for its suitability as a tool for proton beam characterization and QA tasks in the context of MRiPT. In experiments with scanned proton beams its dose rate response, the response homogeneity over the sensitive area, and its effective measurement depth were studied. In addition to this basic detector characterization, tests were performed where the ionization chamber array was used to verify a complex proton treatment plan optimized for two different nozzle distances. Finally, we demonstrated its applicability for MRiPT by measuring square scanned proton fields of different proton energies as well as a patient treatment plan in our in‐beam MR scanner having a 0.32T transverse $B_{0}$ field.

## 2 MATERIAL AND METHODS

### 2.1 Assessment of detector characteristics in scanned proton beams

The ionization chamber array characterized in this work is the Octavius 1500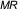 (T10050, PTW Freiburg, Freiburg, Germany).^22^, ^23^, ^24^, ^25^ It has a detector matrix of 1405 air‐vented parallel‐plate ionization chambers, arranged in a chessboard pattern with a diagonal center‐to‐center detector spacing of 7.1mm, resulting in an effective measuring field size of $27\times 27\;{\mathrm{cm}}^{2}$. If line profiles are to be extracted from the measured dose maps, the chessboard dose grid should be interpolated to a uniform dose grid.

**FIGURE 1:**
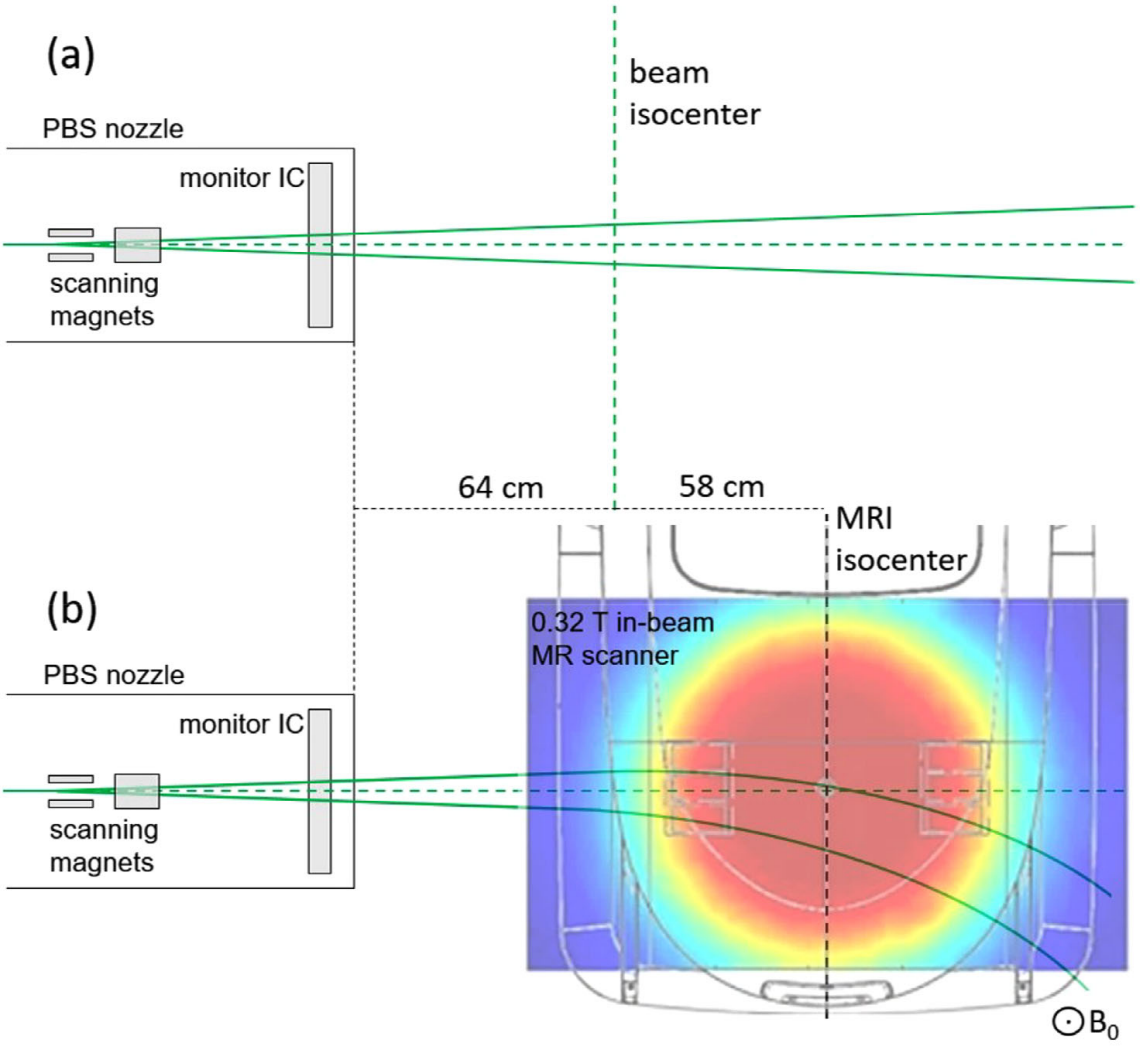
Schematic representation of the PBS nozzle (a) with the beam isocenter indicated and (b) with the in‐beam MR scanner positioned in front of it (transverse magnetic field map of the scanner indicated by the color map). The green lines represent a proton beam path which normally goes straight ahead (a) but gets deflected in the MR magnetic field (b). PBS, pencil beam scanning.

The manufacturer of the Octavius array performs absolute calibration in terms of absorbed dose to water and a homogeneity correction for its ionization chamber arrays in a secondary standard 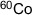 photon beam. For absolute dose measurements in beam qualities different from 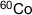 photons (e.g., protons), the manufacturer recommends to cross‐calibrate it against measurements using a reference detector in the user's beam quality. According to the manual, the detector can be safely operated in high magnetic fields (up to 1.5T) and the nominal maximum dose rate is 96Gy/min. All tests performed within this work were done using the dose accumulation mode in measurement range *high*. Experiments were performed in the UPTD experimental room at the horizontal research beamline equipped with a dedicated PBS nozzle.^26^ The isochronous cyclotron based proton therapy system (ProteusPLUS, IBA, Louvain‐la‐Neuve, Belgium) can deliver proton beams in the energy range 70–226.7 MeV as static pencil beams or as PBS plans. Figure 1 shows a schematic representation (top view) of the horizontal beamline with the dedicated PBS nozzle, the beam isocenter and the 0.32T in‐beam MR scanner placed in front of the nozzle.

The reference point for the 2D dose measurements without the MR scanner was the beam isocenter for which the PBS beamline was commissioned. Not shown in Figure 1 are the structural components and the wall of the Faraday cabin in which the MR scanner is located. The device can be moved freely within the experimental room and can be positioned in front of the PBS beamline quickly and reproducibly. The nozzle housing and the Faraday cabin limit how close the scanner can be positioned to the nozzle. With the MR scanner cabin positioned in front of the nozzle, the MRI isocenter is approximately 58cm downstream of the beam isocenter plane. Therefore, the MRI vertical isocenter plane was used as the reference position for measurements inside the magnetic field instead of the beam isocenter plane.

#### 2.1.1 Dose rate response

In a first test, the Octavius 1500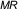 detector was irradiated with proton pencil beams of different currents to see if the detector can handle the high point dose rates. This test is important since the Octavius 1500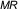 was originally designed for the use in photon beams generated by a linac, which provide beams with dose rates that are much lower than for scanned proton beams. It did not only characterize the dose rate response but at the same time also the range of dose linearity. A linear response over a wide dynamic range is important when highly non‐uniform dose distributions with strong inhomogeneities or sharp dose falloffs should be measured.

The detector array was vertically placed in the beam isocenter plane and centrally aligned by the room lasers to hit the central ionization chamber with a proton pencil beam. An Advanced Markus ionization chamber (TM34045, PTW Freiburg, Freiburg, Germany) read‐out by an electrometer (UNIDOS Tango, PTW Freiburg, Freiburg, Germany) was placed directly behind the Octavius detector, also at the central position, to serve as an independent dose rate monitor. The two detectors were then irradiated with proton pencil beams of varying energy (100–220 MeV) and beam current (0.05–0.5 nA) using the Pristine Beam Tool beam control software (IBA, Louvain‐la‐Neuve, Belgium).

If the dose rate limit of the Octavius 1500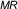 detector is exceeded it switches off automatically. A practical way to reduce the instantaneous dose rate during PBS, in case the beam current cannot be selected directly, is to repaint (or rescan) the scan spot pattern multiple times with a reduced weight.^27^ This technique is typically available to reduce interplay effects for moving tumors.^28^ For the measurements described in this work the repainting method was used to lower the instantaneous dose rates during beam application below the limit of the Octavius 1500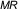 detector.

#### 2.1.2 Response homogeneity

To test the response homogeneity of the Octavius 1500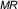 detector, homogeneously scanned fields of $25\times 25\;{\mathrm{cm}}^{2}$ (2.5mm spot spacing, rectangular grid, 1 monitor unit per spot) were irradiated at 100 and 220MeV with the detector centrally positioned at the beam isocenter (Figure 1a). The beam spot sizes (1 σ) were 5mm for 100MeV and 3mm for 220MeV, that is larger than the spot spacing. The dose for both energies (100MeV and 220MeV) was ∼1.5Gy which is a realistic dose for clinical treatment plans. The response homogeneity was quantified by calculating the ratio of the maximum dose to the minimum dose in a region of interest (ROI) covering 80% of the field size. This value can be used as a homogeneity index $HI=D_{max}/D_{min}$ ^29^ and its deviation from the ideal value of 1 indicates the inhomogeneity of the measured dose distribution.

#### 2.1.3 Effective depth of measurement

It is necessary to know the effective measurement depth of the Octavius 1500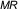 detector if it is to be used for verification of proton treatment plans. This depth must be considered as an offset when exporting lateral dose distributions at the water depths of interest from the treatment planning system. In the literature only one value could be found for photons^30^ and for protons it can be assumed that it is in the same order. However, the effective measurement depth might differ slightly between photon and proton beams due to the radiation quality dependence of the water equivalent thickness (WET)^31^ of the detector housing materials.

The effective measurement depth was determined in two redundant measurements. Pristine Bragg peaks for two proton energies (150MeV and 180MeV) were measured with the Octavius 1500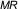 detector and compared with reference Bragg peaks in water (base data of the treatment planning system), measured previously using a Bragg peak chamber (model 34070‐2,5, PTW, Freiburg, Germany), inside a water phantom.^32^ For these measurements, the Octavius 1500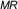 detector was positioned at the beam isocenter (according to Figure 1a) and irradiated with a $10\times 10\;{\mathrm{cm}}^{2}$ scanned field with 2.5mm spot spacing, and polycarbonate plates with known WETs^33^ were successively placed in front of it. For the 150MeV measurement 16 different WETs between 7.0 and 16.5cm were realized and for 180MeV 14 different WETs between 5.8 and 22.0cm were used. In addition, a measurement without plates was performed for both energies. The integrated dose in the central five ionization chambers was analyzed as a function of the WET to obtain the Bragg peaks. The effective measurement depth could then be determined by shifting the depth dose profiles measured with the Octavius detector in order to match the reference Bragg peaks from the measurements in a water phantom (see above). The uncertainty of the determined value could be estimated by comparing the two independent measurements taken at two different proton energies.

#### 2.1.4 Treatment plan QA test without magnetic field

The applicability of the Octavius 1500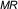 detector for the verification of proton treatment plans was tested for a two‐field plan designed for a patient with esophageal cancer (optimized with RayStation 11B, version 12.0.100.0, dose calculation algorithm: Monte Carlo v5.3, RaySearch Laboratories, Stockholm, Sweden^34^) using the beam model for the beamline in the UPTD experimental room without taking into account the magnetic field. One treatment plan was optimized for the patient being located at the beam isocenter and another one for the same patient, but 58cm further downstream, corresponding to the MRI isocenter plane (see Figure 1). For each treatment plan, the two fields were irradiated with the Octavius 1500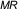 detector positioned either at the beam isocenter (Figure 1a) or at the MRI isocenter (Figure 1b), in a configuration without absorber plate and at two additional water‐equivalent depths by placing plastic plates (IBA MULTIcube phantom) in front. A magnetic field could not be included in these tests because dose calculation and treatment planning in the presence of a magnetic field are still under development.

As in the clinical routine, the individual fields for both plans were recalculated by the treatment planning system in a water phantom and the lateral 2D dose distributions at the planes corresponding to the water equivalent depths of the measurements were exported. For the calculation of these water equivalent depths, also the effective measurement depth of the Octavius 1500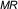 detector determined as described in Section 2.1.3 was taken into account.

The agreement of the calculated with the measured dose distributions for the two fields at the different depths was quantified by performing a 2D gamma analysis using the PTW VeriSoft software (version 8.0.1.0, PTW, Freiburg, Germany). The calculation of the gamma index can be based on a global dose value (typically the dose maximum) or on the local dose values.^35^ Two different gamma criteria were investigated in this work: 3mm/3%(max) (the clinical standard for patient‐specific QA at UPTD with a desired pass rate of >95%) and a more stringent criterion of 2mm/2%(local).

### 2.2 Tests in 0.32 T transverse magnetic field

Since the purpose of this work is to test the suitability of the Octavius 1500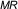 detector for QA tasks in MRI‐guided proton therapy, it was also employed to measure proton dose distributions inside the 0.32 T in‐beam MR scanner (setup as shown in Figure 1b). For these measurements, the Octavius detector was positioned in the MRI isocenter plane and irradiated with $15\times 15\;{\mathrm{cm}}^{2}$ square fields of three different proton energies (100, 160, and 220MeV) as well as the two fields of the patient treatment plan optimized for the MRI isocenter plan (without considering the magnetic field in dose calculation). For the square fields, the spot distance at the MRI isocenter plane was 2.5mm irradiated uniformly with 1 monitor unit per spot.

## 3 RESULTS

### 3.1 Detector characteristics

#### 3.1.1 Dose rate response

**FIGURE 2:**
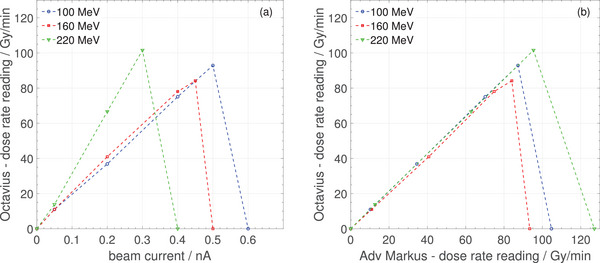
Dose rate response of the PTW Octavius 1500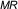 ionization chamber array tested with proton pencil beam energies of 100 MeV (blue circles), 160 MeV (red squares) and 220 MeV (green triangles). (a) Octavius dose rate reading as a function of beam current, and (b) as a function of dose rate measured with an Advanced Markus chamber behind the Octavius detector.

Figure 2a shows the dose rate reading as a function of the nozzle beam current for three energies (100, 160, and 220MeV). It can be observed that the dose rate reading increases linearly until a certain beam current is reached where the measurement is interrupted and the reading is zero. This interruption occurs at readings above 80–100 Gy/min, which is in good agreement with the nominal limit of 96Gy/min stated in the manual of the detector.^22^ Furthermore, the response curve for 220MeV does not overlap with the other two energies. The beam current alone is not a suitable measure in this case because the local dose rate in a proton pencil beam also depends on the size and shape of the beam spot and the proton energy loss. Lower proton energies have larger beam spots but a higher linear energy transfer. In Figure 2b the beam current on the *x*‐axis is replaced by the dose rate reading from the Advanced Markus chamber mounted behind the Octavius 1500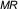 detector and in this plot the three curves are overlapping.

#### 3.1.2 Response homogeneity

**FIGURE 3:**
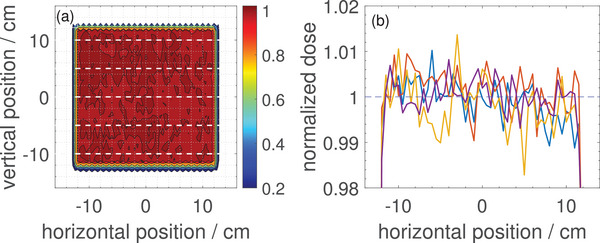
(a) Normalized 2D dose distribution of a $25\times 25\;{\mathrm{cm}}^{2}$ scanned 100MeV proton field measured with the Octavius 1500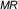 ionization chamber array, and (b) horizontal profiles at 5cm equidistant vertical positions over the field, indicated with white dashed lines in (a).

Figure 3a shows the measured lateral 2D dose distribution for the example of a scanned field of 100MeV protons normalized to the mean value in a ROI covering 80% of the field. Figure 3b shows line profiles at different representative vertical positions along the field (distributed equidistantly with 5cm distance). The fluctuation in these profiles is in the order of ±1.5% at maximum. As found in separate experiments where the Octavius detector was intentionally misplaced by a few millimeters this structure on the profiles moves together with the detector. Therefore, it is clearly a response inhomogeneity of the detector and not an inhomogeneity of the beam.

The homogeneity index was 1.034 for the 100MeV field and 1.038 for the 220MeV field (not shown in Figure 3). The uncertainty of these HI values was estimated to be about 0.002 by slightly varying the ROIs.

#### 3.1.3 Effective depth of measurement

**FIGURE 4:**
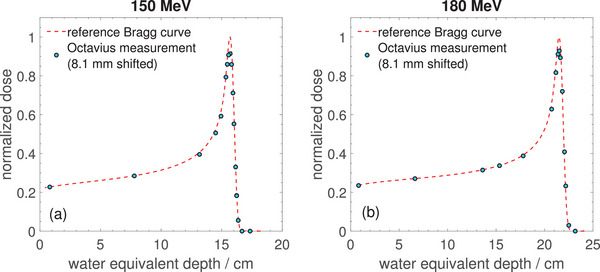
Bragg peaks for 150 MeV (panel (a)) and 180 MeV (panel (b)) proton beams measured with the Octavius 1500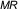 detector using polycarbonate plates taking into account the effective depth of measurement of the detector.

Figure 4a,b shows the normalized depth dose profiles for 150MeV and 180MeV protons measured with the Octavius 1500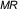 detector compared with reference Bragg curves. The Octavius data was shifted in depth by an offset value of 8.1mm which is the effective measurement depth, mainly due to the detector housing in front of the ionization chambers. The uncertainty of this value is estimated from the independent measurements performed at two different energies, also taking into account the uncertainty of the WETs of the plates used, to be about 0.2mm. Notably, the Bragg peak heights measured with the Octavius detector using the polycarbonate plates are 7–8% lower than in the reference curves measured in water.

#### 3.1.4 Treatment plan QA test without magnetic field

**FIGURE 5:**
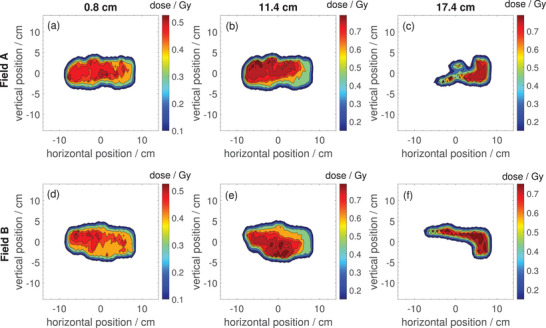
2D dose distributions for two fields of proton treatment plan for a patient with esophageal cancer optimized for the beam isocenter measured with the Octavius 1500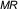 detector at water equivalent depths of 0.8, 11.4 and 17.4cm.

Figure 5 shows the measurements for the two radiation fields A and B for the esophageal cancer treatment plan optimized for the beam isocenter at three different water equivalent depths (0.8, 11.4 and 17.4cm). The depths listed include the effective measurement depth determined in Section 3.1.3, rounded down to 8mm due to the limited dose grid size when re‐calculating the dose distributions. A plan for the same patient optimized for the MRI isocenter plane was measured as well using the same configurations (not shown here but in Section 3.2.2).

Table 1 shows the pass rates for the 2D gamma analysis comparing the measured dose distributions with the calculated distributions from the treatment planning system, both normalized to the dose at the same point.

**TABLE 1:** Gamma pass rate for the two radiation fields of proton treatment plans for a patient with esophageal cancer (one optimized for the beam isocenter and the other one for the MRI isocenter plane) as calculated by the treatment planning system compared to measurements using the Octavius 1500 detector at water equivalent depths of 0.8, 11.4, and 17.4cm.

	Water equivalent depth		
Beam isocenter	0.8 cm	11.4 cm	17.4 cm
**Field A**	3mm/3%(max):100%	100%	100%
	2mm/2%(local):96.4%	97.2%	98.1%
**Field B**	3mm/3%(max):99.6%	100%	99.4%
	2mm/2%(local):92.8%	97.1%	96.8%

	Water equivalent depth		
MRI isocenter	0.8 cm	11.4 cm	17.4 cm
**Field A**	3mm/3%(max):100%	100%	100%
	2mm/2%(local):100%	99.4%	93.8%
**Field B**	3mm/3%(max):100%	100%	100%
	2mm/2%(local):100%	96.7%	96.7%

*Note*: Pass rates for two different gamma criteria (3mm/3%(max) and 2mm/2%(local)) are shown.

### 3.2 Tests in 0.32 T transverse magnetic field

#### 3.2.1 Square fields

**FIGURE 6:**
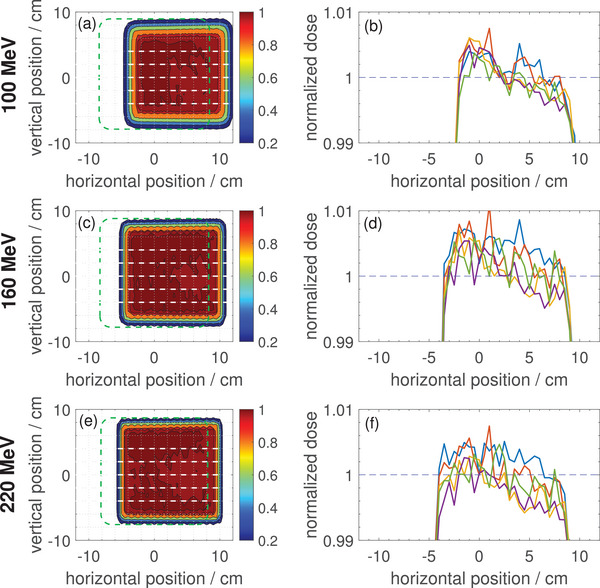
(a, c, e) 2D dose distributions in beam's‐eye‐view for $15\times 15\;{\mathrm{cm}}^{2}$ fields scanned with three different proton energies (100, 160, and 220MeV) measured with the Octavius 1500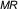 detector inside the 0.32T in‐beam MR scanner at the MRI isocenter plane. The green dashed squares indicate the position of the field edges without a magnetic field. (b, d, f) Horizontal profiles at selected vertical positions (equidistantly distributed with 2cm distance, indicated with white dashed lines in (a, c, e)). MRI, magnetic resonance imaging.

Figure 6 shows 2D dose distributions and horizontal profiles for a $15\times 15\;{\mathrm{cm}}^{2}$ field irradiated with three different proton energies measured with the Octavius 1500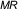 detector positioned at the MRI isocenter plane (Figure 1b). The location of the measured field edges without the magnetic field are indicated by the green dashed lines in the left panels.

As expected, it can be observed that the fields are shifted laterally (to the right in beams‐eye‐view) due to the proton beam deflection in the 0.32T transverse magnetic field. The shift decreases with increasing energy (3.7cm for 100MeV, 2.9cm for 160MeV, 2.3cm for 220MeV). A small dose gradient (∼1% over 10cm) can be recognized in the 2D plots and horizontal dose profiles. The absolute dose readings in the MR scanner are consistent with measurements of the same radiation fields without magnetic field within ∼1%.

#### 3.2.2 Patient treatment plan

**FIGURE 7:**
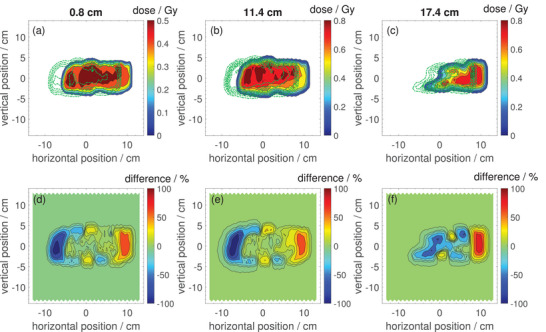
(a, b, c) 2D dose distributions in beam's‐eye‐view for the first field (field A) of a proton treatment plan for a patient with esophageal cancer at water equivalent depths of 0.8, 11.4, and 17.4cm measured with the Octavius 1500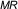 detector inside the 0.32T in‐beam MR scanner. The green dot‐dashed lines are the isodose lines for the same treatment plan measured at the MRI isocenter plane without the presence of the magnetic field. (d, e, f) Relative difference between the dose distributions measured inside the MR scanner compared to those measured without presence of the magnetic field. MRI, magnetic resonance imaging.

**FIGURE 8:**
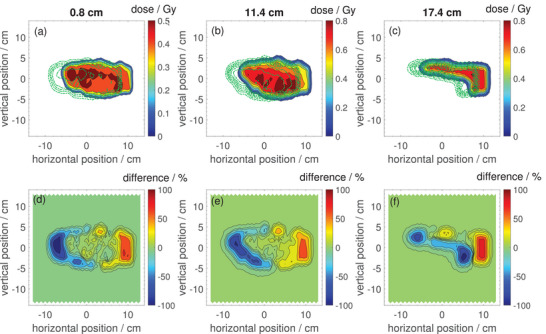
(a, b, c) 2D dose distributions in beam's‐eye‐view for the second field (field B) of a proton treatment plan for a patient with esophageal cancer at water equivalent depths of 0.8, 11.4, and 17.4cm measured with the Octavius 1500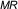 detector inside the 0.32T in‐beam MR scanner. The green dot‐dashed lines are the isodose lines for the same treatment plan measured at the MRI isocenter plane without the presence of the magnetic field. (d, e, f) Relative difference between the dose distributions measured inside the MR scanner compared to those measured without presence of the magnetic field. MRI, magnetic resonance imaging.

Figures 7 and 8 show measured 2D dose distributions for the same treatment plan for a patient with esophageal cancer (optimized for the MR isocenter plane) that was employed for the patient specific QA test in Section 3.1.4, but now in presence of the 0.32T magnetic field of our in‐beam MR scanner.

The upper panels (a–c) show the dose distributions for the same three depths as studied in Section 3.1.4 as color maps. The green dash‐dotted lines overlayed on the color maps are the isodose lines of the respective dose distibutions measured without the presence of the magnetic field. The lower panels (d–f) show difference maps between the dose distributions measured with and without presence of the magnetic field.

## 4 DISCUSSION

The Octavius 1500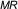 ionization chamber array is a well‐established tool for dosimetric QA tasks at low‐field and high‐field MR‐linacs.^23^, ^24^, ^25^ In this work, different characteristics of the detector to assess its suitability for measurements in scanned proton beams were studied in experiments as described in the previous sections. An additional focus was put on its performance in the presence of a 0.32T transversal magnetic field of our in‐beam MR scanner.

In Figure 2 the dose rate dependence of the Octavius 1500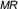 is shown. The maximum dose rate that can be measured before the measurement is interrupted is in good agreement with the nominal dose rate limit of 96Gy/min given in the manual.^22^ The practically usable range of dose rates can be defined as 80Gy/min and below. The good linearity for dose rates below 80Gy/min is in agreement with the dose and dose rate linearity deviation smaller than 0.5% stated in the manual.^22^ Scanned proton beams may occasionally exceed this range of dose rates, which should be avoided when using the Octavius 1500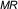 detector. Especially for fields or treatment plans using high proton energies with sharp beam spots this can be an issue. Depending on the field, 5–6 repaints per energy layer were necessary in order to lower the instantaneous dose rate below the limit of the Octavius 1500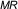. It should be noted that the relation of the instantaneous dose rate and the number of repaints observed in our experiments was not linear and it is expected to depend strongly on the exact time structure of the scanned beams of the proton therapy system used.

The obtained homogeneity values 1.034 for the homogeneously scanned 100MeV field and 1.038 for the 220MeV field would be sufficient for application of the Octavius 1500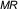 array for QA of patient treatment plans according to the requirements in our proton therapy center (see gamma criteria in Section 3.1.4). The slightly larger HI value observed for the 220MeV field compared to 100MeV might be associated with the smaller beam spot size. The majority of the fluctuations observed in the measurements are likely associated with the response of the ionization chamber array rather than being actual field inhomogeneities in the scanned beam. The field homogeneity is actively controlled during the PBS process and checked regularly for similar fields during machine QA using a LynxPT detector and the observed inhomogeneity is typically below 1%. The response map for the Octavius 1500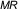 ionization chambers is calibrated by the manufacturer using a 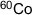 photon beam prior to delivery to the user (see Section 2.1). According to the Octavius manual,^22^ after application of the 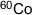 calibration map the response of the individual chambers can still deviate up to ±1.5%. This is in the same order like the measured homogeneity indices and comparable to the fluctuations on the dose profiles shown in Figure 3b.

The effective measurement depth of the Octavius 1500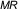 for protons was determined as 8.1±0.2mm which compares well with the value of 8.7±0.2mm reported by Stelljes et al.^30^ for photon beams. The small difference is probably due to differences between photon and proton radiation physics and therefore slightly different WETs of the detector housing materials for the different radiation qualities. As visible in Figure 4, the shape of the Bragg curves is also well reproduced by the Octavius measurements, except for an underestimation of the Bragg peak height by 7%–8%. This discrepancy could be due to averaging effects in the ionization chambers or partly also due to the non‐water‐equivalence of the polycarbonate plates used for energy degradation.

Figure 5 shows measured 2D dose distributions for a two field proton treatment plan optimzed for the beam isocenter without presence of the in‐beam MR scanner. The radiation fields have a complex shape, as expected from a clinical treatment plan. Especially the largest depth provides a good test of the suitability of the Octavius 1500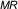 detector for verification and QA purposes because of the sharp dose gradients present at the distal field edge. Table 1 shows the gamma pass rate for the comparison of RayStation dose calculation versus Octavius measurements for this plan (optimized for the target being located in the beam isocenter plane) as well as an equivalent plan optimized for the MR isocenter plane which is located 58cm further downstream (see Figure 1). For the 3mm/3%(max) criterion, all cases passed the gamma test (gamma pass rate >95%). The pass rate for the 2mm/2%(local) criterion was above 95% in five out of six cases for the beam isocenter and MR isocenter, respectively. These tests without magnetic field confirm that the Octavius 1500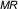 detector is suitable for such kind of patient‐specific QA measurements in scanned proton beams. Furthermore, it confirms that the dose calculation algorithm in RayStation used for optimization of the treatment plan is accurate (as known from various benchmark experiments^34^).

Having demonstrated the suitability of the Octavius 1500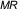 detector for patient‐specific QA in proton therapy without the presence of an external magnetic field, the question remains whether this is also the case in the presence of a transverse MR magnetic field. Since proton dose calculation in the presence of magnetic fields in RayStation is still under development and thus no treatment plan optimization in magnetic fields is established yet, we measured standard QA square patterns in the magnetic field and also measured the patient treatment plan that was optimized for the MR isocenter without presence of the magnetic field inside the MR scanner in order to quantify the effect of the magnetic field on the plan quality.

Figure 6 shows the square fields for 100, 150, and 220MeV protons measured at the MR isocenter plane. The energy dependent shift of the fields matches within 2–3 mm with a fit function based on experimental data taken with EBT‐3 films in a previous study (3.4cm for 100MeV, 2.7cm for 150MeV, and 2.1cm for 220MeV ^36^). Comparing the isodose lines with the grid lines, one can also notice (especially for the 100MeV measurement) that the square fields are slightly deformed into trapezoidal fields. On the left side the field edges are slightly compressed while on the right side they are slightly stretched. This effect is well‐known from Monte Carlo simulations^37^ and measurements^36^ and can be understood by considering the proton transport through the magnetic field of the MR scanner including the inhomogeneous fringe field. Despite this deformation of the radiation fields, the lateral dose profiles are relatively flat as visible in Figure 6b,d,f. The small dose gradient (∼1% over 10cm) is probably due to local changes in proton fluence as a result of the field deformation. The ability of the Octavius measurements to detect such small effects of the magnetic field on the dose distributions will make the Octavius 1500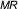 detector a very helpful tool for the future validation and commissioning of the proton dose calculation algorithms taking into account the magnetic field, that are currently under development.

Figures 7 and 8 show 2D dose distributions for the treatment plan optimized for the MR isocenter measured inside the in‐beam MR scanner (color plots) and without the presence of the magnetic field (green dash‐dotted isodose lines). The shape of the dose distributions inside the MR scanner looks comparable to those measured without the presence of the magnetic field, but especially the strong lateral shift due to the proton deflection would lead to large regions with almost 100% under‐ and over‐dosage, respectively. Therefore, it is clear that a compensation for these effects is necessary for proton treatments inside an in‐beam MR scanner. The required number of repaints per energy layer (i.e., the dose rate limit) was identical for the measurements inside the MR scanner compared to the measurements without magnetic field. From this observation one can conclude that the switching off behavior at high dose rates as characterized in Section 3.1.1 is not affected by the magnetic field.

The characterization of the Octavius 1500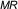 ionization chamber array presented in this work facilitates its application for verification of 2D dose distributions in MRI‐guided proton therapy, for example in the framework of patient‐specific QA. Such measurements should be performed inside the MR scanner in order to verify the correct prediction of the effect of its static magnetic field on the proton dose distributions by the treatment planning system. The proton beams experience a Lorentz‐force induced, beam energy‐dependent deflection in the magnetic field of the MR scanner which bends them on a circular trajectory. If the Octavius 1500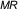 detector is positioned with its surface perpendicular to the nominal beam axis (horizontal dashed line in Figure 1b), as it would be positioned by default for measurements without magnetic field, the protons impinge on the detector with an angle and pass through a slightly larger effective thickness of the detector housing materials. Since this angle depends on the proton energy, a tilt of the detector could only partially compensate for this. Therefore, it is recommended to position the Octavius 1500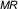 detector for measurements inside the MR scanner perpendicular to the nominal beam axis, as it would be positioned for measurements without magnetic field, and to consider this variation of the WET in front of the active volume of the ionization chambers due to the angle of the proton beams in the re‐calculation by the treatment planning system. When modelling the Octavius 1500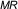 detector as a block of water oriented perpendicular to the nominal beam axis, and calculating the dose at 8.1mm depth from the surface (the effective depth of measurement determined without magnetic field, see Section 3.1.3), this effect is inherently considered in the dose calculation. If the Octavius 1500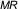 ionization chamber array should not only be used for verification of relative dose distributions but also for absolute dose checks, the manufacturer recommends a cross‐calibration against a reference detector in the user's beam quality.^22^ Because the response of ionization chambers can be slightly modified (typically less than 1%) by the presence of a magnetic field,^16^ the cross‐calibration with proton beams should be performed inside the MR scanner in order to take those effects correctly into account.

## 5 SUMMARY AND CONCLUSION

The Octavius 1500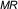 ionization chamber array was experimentally characterized using scanned proton beams and tested for its applicability as a dosimetric tool for beam characterization and QA for MRI‐guided proton therapy.

For scanned proton therapy plans with intense dose spots, the dose rate limitation of the detector can be circumvented by reducing the beam current and/or by repainting of the dose spot patterns. The dose response homogeneity to homogeneous proton fields was found to be sufficient. The effective measurement depth was determined to be 8.1±0.2mm. A QA test for a realistic clinical patient treatment plan was successful and the detector was shown to be operational and provide reasonable measurements in the presence of the 0.32T magnetic field of our in‐beam MR scanner. It was even able to resolve the deformation of scanned square proton fields by magnetic field effects which makes it a valuable tool for the commissioning of treatment planning and dose calculation in the presence of magnetic fields. Measurements of a patient treatment plan inside the MR scanner and comparison with corresponding measurements without the scanner allowed a detailed assessment of the magnetic field effects on the dose distributions, which demonstrates the potential suitability of the Octavius 1500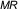 detector for patient‐specific QA.

## CONFLICT OF INTEREST STATEMENT

The authors declare no conflicts of interest.
